# The Origin and Evolution of Ribonucleotide Reduction

**DOI:** 10.3390/life5010604

**Published:** 2015-02-27

**Authors:** Daniel Lundin, Gustav Berggren, Derek T. Logan, Britt-Marie Sjöberg

**Affiliations:** 1 Department of Biochemistry and Biophysics, Arrhenius Laboratories, Stockholm University, SE-106 91 Stockholm, Sweden; E-Mails: gustav.berggren@kemi.uu.se (G.B.); britt-marie.sjoberg@dbb.su.se (B.-M.S.); 2 Department of Biochemistry and Structural Biology, Lund University, Box 124, SE-221 00 Lund, Sweden; E-Mail: derek.logan@biochemistry.lu.se

**Keywords:** ribonucleotide reductase (RNR), RNA/RNP world, DNA building blocks, protein evolution, redox chemistry, radical chemistry, deoxyribonucleotides, primordial hydrogen atom abstraction, structural phylogenetics, allosteric regulation

## Abstract

Ribonucleotide reduction is the only pathway for *de novo* synthesis of deoxyribonucleotides in extant organisms. This chemically demanding reaction, which proceeds via a carbon-centered free radical, is catalyzed by ribonucleotide reductase (RNR). The mechanism has been deemed unlikely to be catalyzed by a ribozyme, creating an enigma regarding how the building blocks for DNA were synthesized at the transition from RNA- to DNA-encoded genomes. While it is entirely possible that a different pathway was later replaced with the modern mechanism, here we explore the evolutionary and biochemical limits for an origin of the mechanism in the RNA + protein world and suggest a model for a prototypical ribonucleotide reductase (protoRNR). From the protoRNR evolved the ancestor to modern RNRs, the urRNR, which diversified into the modern three classes. Since the initial radical generation differs between the three modern classes, it is difficult to establish how it was generated in the urRNR. Here we suggest a model that is similar to the B_12_-dependent mechanism in modern class II RNRs.

## 1. Introduction

**Figure 1:**
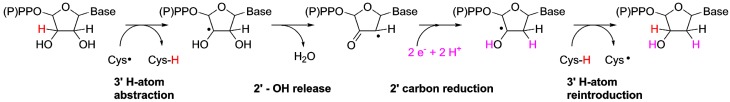
Ribonucleotide reduction takes place in four basic steps, the first of which involves activation of the substrate through abstraction of a hydrogen atom at the 3' position of the ribose. Subsequently, the 2' OH-group leaves as water, the substrate is reduced with two electrons and the initially abstracted 3' hydrogen is returned to the substrate to form the complete product. Adapted from Torrents *et al.* 2008 [1].

DNA is the genetic material in all cellular organisms plus many viruses. DNA’s building blocks, deoxyribonucleotides (dNTPs), are always synthesized by reduction of ribonucleotides (either NTPs or NDPs), the building blocks of RNA. The reaction is catalyzed by RNR via a chemically demanding mechanism that involves activation of the substrate by abstraction of the H-atom from the 3' position, performed by a protein-derived cysteinyl free radical (Figure 1). This activates the substrate so that the OH-group can leave from the 2' position as water. Subsequently, the substrate is reduced and the initial cysteinyl radical is reformed by H-atom abstraction performed by the substrate, completing the reaction. In modern RNRs, the essential cysteinyl radical is generated by three profoundly different mechanisms, forming the foundation for how modern RNRs are divided into three homologous classes.

Many aspects of ribonucleotide reduction are profoundly interesting from an evolutionary point of view: (i) The origin of the reaction, because of its association with the transition from RNA- to DNA-encoded genomes (Section 2.2); (ii) how the three classes showcase how evolution has discovered some of the most potent uses for metals in redox chemistry (Section 3.3 and Section 4.3); (iii) the ecological implications of the different oxygen and metal requirements of the three radical generation mechanisms (Section 4.3); and (iv) the finely tuned allosteric substrate specificity and overall activity regulation of RNRs (Section 3.5 and Section 3.6), which ensures that dNTP pools are in balance to avoid increased mutation rates during replication.

## 2. Origin of Ribonucleotide Reduction

How and when ribonucleotide reduction evolved is a question that is intimately associated with the transition from the RNA world to the modern RNA + protein + DNA world, since it is the only known *de novo* mechanism for dNTP synthesis. This does not rule out other mechanisms for dNTP synthesis during evolutionary time. Recently, the reverse aldolase reaction was put forward as a candidate alternative pathway [2,3,4]. This is an interesting hypothesis, but here we wish to propose a model for how ribonucleotide reduction could have originated in the RNA + protein (RNP) world by a mechanism closely resembling the modern one (Figure 1). To emphasize that our model should not be seen as an exclusive alternative to other pathways, we wish to stress that an early source of deoxyribonucleotides as a result of e.g., ribozymes performing the reverse aldolase reaction may have preceded ribonucleotide reduction to be later succeeded by the precursor of modern RNR chemistry.

### 2.1. The RNP World

**Figure 2:**
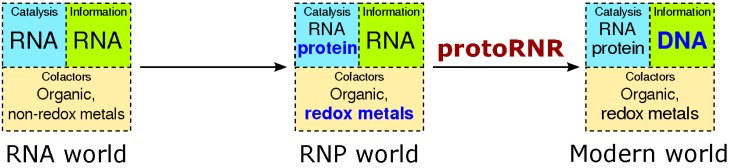
The transition from the RNA world to the RNP world and, by the action of the protoRNR, to the modern RNA + protein + DNA world. Evolutionary novelties are marked in bold blue text. Whereas non-redox active divalent metals in ribozymes are common, metals catalyzing electron transfer in ribozymes are much more rare (see main text).

The stage for our model is one where the first steps from a pure RNA world (see [5] for a recent review) towards an RNP world have been taken. We assume translated proteins have originated, acting as assembly points for redox-active metals as well as RNA world cofactors (ribonucleoside-derived carbon radicals). Besides working as assembly points, proteins isolate cleavage-sensitive RNAs from strong redox-potentials in metals and organic radicals (Figure 2).

It is difficult to estimate the coding capacity of an RNP world organism and how its genetic material was used in terms of number and length of genes. We may assume, however, that there were strict limits on coding capacity and its usage (but see [6] for how an early origin of proofreading and repair activities of RNA polymerase could ameliorate the situation). Strict limitations on coding capacity will thus impose selection against innovation as the appearance and fixation of a new function must not only be selectively advantageous by increasing an individual’s fitness, but must also (i) replace an existing function; (ii) improve an existing function; or (iii) by itself or in conjunction with other processes increase the useful coding capacity of the organism. The latter has been suggested as an escape from the Eigen limit on genome size [7,8] by a ratchet-like process, with selection acting on replication-fidelity that increases the maximum genome size [9]. We suggest that ribonucleotide reduction evolved by selection for increased pools of deoxyribonucleotides, as DNA was evolving to become the coding material of choice. The selectional advantage behind ribonucleotide reduction would thus be of type iii, *i.e.*, increase of the coding capacity. Irrespective of the details of the process that led to DNA, we assume it was a stepwise process. It cannot have anticipated the advantages of the modern situation with a DNA genome in the hands of a complete set of replicating and transcribing enzymes. We thus assume that ribonucleotide reduction evolved in parallel with a stepwise transition to DNA as coding material.

Cofactors, in particular metals, minerals and nucleoside derivatives, are believed to have played an important role in catalysis in the RNA/RNP worlds. This is supported by the fact that in many modern enzymes, the reactive part in catalysis is not played by the protein chain but by cofactors, many of which have a likely origin in the RNA world (see [10] and references therein). Biological polymers—first RNA molecules, later proteins—were in many cases mere scaffolds for cofactors and binding sites for substrates. Potentially, chemistries could transfer from ribozyme to protein just by transfer of metals or other cofactors as suggested by Yarus [11]. As a point in case, ribonucleotide reduction has been suggested to have been catalyzed by a ribozyme [12], but the suggestion has been met with considerable skepticism because of potential problems with free radicals in RNA molecules [13]. Hsiao *et al.* [14] have reported redox catalysis by iron in a ribozyme and Hamm *et al.* [15] have reported that Mn^2+^ bound to the Hammerhead ribozyme is more active than Mg^2+^ and that activity is affected by radical scavengers. These are indeed intriguing observations, suggesting that RNA may be more resilient to radicals and strong redox metals than hitherto assumed. However, most metals bound to ribozymes are redox inert divalent ions, commonly Mg^2+^ [16]. Proteins are more resilient than RNA to strong redox-active compounds and we thus lean towards a protein being a more likely polymer scaffold for early ribonucleotide reduction. Indeed, many modern enzymes, including class I and III RNRs, harbor stable amino acid radicals. Hence, the appearance of proteins in the RNA world likely unleashed a potential for more potent chemistry powered by redox-active metals, by shielding RNA from strong redox potentials and free radicals. Early folds, such as the TIM barrel [17], contain enzymes performing this type of chemistry. As an example, the TIM barrel radical-SAM enzymes catalyze numerous reactions utilizing potent radical chemistry [18], and allow e.g., difficult oxidation reactions to proceed under anaerobic conditions [19].

### 2.2. The ProtoRNR

The appearance of powerful metal-catalyzed redox chemistry was a crucial step for the origin of ribonucleotide reduction. Arguably the most difficult step during RNR-catalyzed ribonucleotide reduction is the initial activation of a chemically unreactive C–H bond, and the only pathway known to date involves a cysteinyl radical capable of abstracting the 3’ H-atom from the ribose moiety (Figure 1). This initial radical is in turn always generated via a metal-containing cofactor. Our proposal for a prototypical RNR, the protoRNR, builds on the modern reaction mechanism, and cofactors and chemistries likely present in early life. We thus suggest a protein with a metal/nucleosyl cofactor reactive enough to perform H-atom abstraction from an unreactive C–H bond. Such an early protein is unlikely to have been specific for RNR activity, but would rather have acted on a wide range of different substrates. However, in the following we will focus on its potential mechanism for ribonucleotide reduction.

#### 2.2.1. Reaction Mechanism of the ProtoRNR

Modern enzymes display a wide range of cofactors for performing H-atom abstraction chemistry. The most common intermediate under anaerobic conditions is the 5' deoxyadenosyl 5' radical (dAdo•), *i.e.,* a radical positioned on the dehydroxylated 5' carbon of the ribose ring of an adenosine. This species is generated either via homolytic cleavage of adenosylcobalamin (AdoCbl) or from *S*-adenosylmethionine (AdoMet) by single electron injection from an iron-sulfur cluster [19,20,21]. These cofactors are among those considered the most ancient and are still observed in modern class II and III RNRs. In class II RNRs the electron hole on AdoCbl-derived dAdo• is transferred to the 3' position of the substrate nucleotide via a cysteinyl radical intermediate. In class III the AdoMet-derived dAdo• first generates a stable glycyl radical in the RNR proper. Subsequently, the radical is transferred from the glycyl to a proximal cysteine, which in turn performs the H-atom abstraction from the substrate. Interestingly, in both classes, ribonucleoside derivatives are thus acting both as radical instigators (dAdo•) and substrates (either NTPs or NDPs).

In this context it should also be noted that while the biosynthesis of both iron-sulfur clusters and metalloporphyrins (e.g., AdoCbl) requires complex machineries in modern organisms [22,23], iron-sulfur clusters can form spontaneously *in vitro* from relatively simple starting materials [24,25] and abiotic porphyrin synthesis has been suggested [26]. Moreover, both cobalt and iron are considered to have been bioavailable to a much greater extent prior to the great oxygenation event than they are today (see [27] and references therein).

The chemical nature of the first synthetically useful species capable of H-atom abstraction will obviously be difficult to elucidate. Nevertheless, following the reasoning outlined above, one can envision a protoRNR capable of reductive formation of dNTPs based on mechanistic principles still observed in modern RNRs. Inside a partly open oligopeptide, the substrate nucleotide is recognized by relatively simple H-bonding interactions at the N-terminus of one or more α helices [28], without substrate specificity based on the identity of the nucleobase. The substrate is placed in the vicinity of a metal center to which a second nucleoside moiety, e.g., AdoMet or AdoCbl, is covalently bound. The metal complex is responsible for the generation of the nucleosyl radical, e.g., dAdo•, as observed in radical-SAM or AdoCbl enzymes. The generated highly reactive dAdo• in turn abstracts the 3’ H-atom from the ribose moiety of the substrate, either directly or proceeding via an intermediate amino acid-centered radical. The latter alternative is most similar to the mechanism observed in modern RNRs, but is not strictly required.

**Figure 3:**
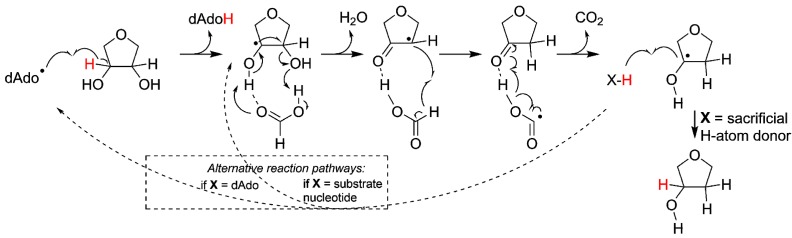
Simplified reaction mechanism for the reduction of ribonucleotides (for simplicity only the ribose moiety is shown). The reaction is initiated through abstraction of the 3'-H atom by e.g., dAdo•, followed by rearrangement and reduction of the 2'-C position. During the reaction formate serves the role of both base and H-atom donor. We suggest three ways of reforming the 3' C–H bond: (i) e.g., formate donates a second H-atom, terminating the reaction; (ii) the H-atom is abstracted from a second ribonucleotide; or (iii) the H-atom is reabstracted from dAdoH. The two latter possibilities (marked with dashed lines) would make the system catalytic, but are less likely from kinetic and thermodynamic viewpoints, respectively.

Following the initial 3' H-atom abstraction we have arrived at the first intermediate of the reaction (Figure 3). However, ribonucleotide reduction is not only dependent on H-atom abstraction but also requires a source of protons and reducing equivalents. While most RNRs today utilize glutaredoxins or thioredoxins as terminal reductants, there are oxygen sensitive forms of RNR that utilize a small molecule, formate, as electron and proton donor [29]. Thus, in line with the latter, once the reaction has been initiated in the protoRNR it could proceed via electron and proton transfer from a small molecule like formate. Once the radical has returned to the 3’ carbon (Figure 3, right), the formation of the final product can proceed via H-atom abstraction from (i) a sacrificial H-atom donor (e.g., a second equivalent of formate; Figure 3, solid arrow); (ii) another nucleotide (Figure 3, right dashed arrow), resulting in a radical chain reaction; or (iii) dAdoH, in which scenario the catalytic cycle is closed as the H-atom is reabstracted from dAdoH to form the product deoxyribonucleotide while dAdo• is regenerated (Figure 3, left dashed arrow). The latter reaction would be thermodynamically unfavored [30], nevertheless such a catalytic role of AdoMet/dAdo• has been suggested in for example radical-SAM mutases such as lysine 2,3-aminomutase and spore photoproduct lyase [19,31], and it is reminiscent of the cobalamin-stabilized dAdo• observed for class II RNR.

#### 2.2.2. The Three-Dimensional Structure of the ProtoRNR Protein

When considering what type of three-dimensional structure the protoRNR could have had, folds of the β/α type, e.g., the 8-stranded TIM β/α barrel and the 5-stranded β/α sandwich flavodoxin-like folds, are of considerable interest. Among modern enzymes with the TIM β/α barrel and flavodoxin-like folds, we find several examples of chemistry resembling what we propose above. For example, the radical-SAM enzymes belong to the TIM β/α barrel fold. They harbor an Fe_4_S_4_-cluster covalently bound to AdoMet, and the dAdo• is formed by reductive cleavage [19]. The class III RNR activase and other glycyl radical enzyme (GRE) activases belong to this superfamily and perform H-atom abstraction from a specific glycine residue. Other members of the same fold—e.g., methylmalonyl-coenzyme A mutase [32]—bind vitamin B_12_ in the form of AdoCbl, from which a dAdo• is also generated. Similarly, methionine synthase that has a flavodoxin-like fold also generates dAdo• from an AdoCbl moiety. Not only does the chemistry performed by these enzymes resemble what we propose for the protoRNR, but they are also considered ancient folds on the basis of phylogenetic evidence, domain distribution and the diversity of functions and cofactors [17,33,34,35]. Furthermore, it was recently proposed that the TIM β/α barrel and the flavodoxin-like folds have a common evolutionary origin, potentially from a (β/α)_2_ structural element [36] and a recent analysis suggests evolutionary relatedness between many α/β proteins [37].

#### 2.2.3. The ProtoRNR: An Unspecific Metal-Catalyzed Radical Enzyme

In summary, we propose that ribonucleotide reduction was *one* of several reactions catalyzed by an early, unspecific enzyme, here called the protoRNR because of our focus on dNTP synthesis. Assuming an evolutionary process based on tinkering, *i.e.*, modification of present components rather than inventions from scratch, our model of the protoRNR tries to deviate as little as possible from what is observed in modern biochemistry. In a fold possibly made up of several β/α motifs, a metal center instigates a carbon radical on a nucleoside with the potential to activate substrates via H-atom abstraction to perform reactions that were likely unreachable in a pure RNA-based world. In modern anaerobes, many of the most demanding redox reactions are performed by enzymes that at their base utilize dAdo•. Importantly, the model is in principle testable, at least in its separate details, starting e.g., from a modern radical-SAM enzyme.

## 3. Origin of the UrRNR

**Figure 4:**
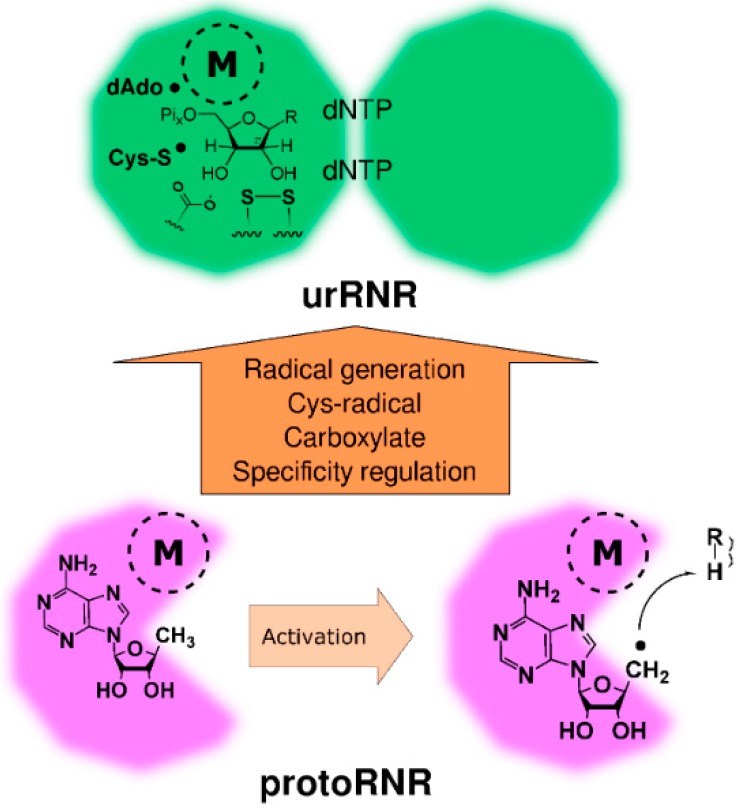
Activation of the protoRNR by formation of a nucleosyl radical (bottom) and evolution of the protoRNR to the urRNR (top). The protoRNR is a general substrate activator—shown here with an R-H substrate—acting e.g., via H-atom abstraction like in RNR (Section 2.2). Some of the generality may have remained in the urRNR, but we have chosen to draw a nucleotide as substrate in the urRNR (key members of the H-atom transfer pathway are shown in radical form). The “M” in a dashed circle denotes the metal center of the protoRNR and urRNR, respectively. See main text and Table 1 for a description of the urRNR.

In the case of the protoRNR we dealt with evolution prior to the common ancestor of modern enzymes, forcing us to rely on general principles informed by modern RNRs and other enzymes performing reactions involving free radicals. We are on firmer ground when we now turn to the later evolution of ribonucleotide reduction and in particular to the origin of the common ancestor of modern RNRs, the urRNR, and how it diverged into the modern three classes. However, lacking fossils, evolutionary reconstructions of proteins are limited to inferences based on modern proteins, which allow us to reconstruct the common ancestor of modern proteins by observing the traits of modern proteins. Following the principle of parsimony, urging us to propose the minimum complexity in the process leading to the current state, we will reconstruct the urRNR from commonalities found between modern RNRs. Bearing in mind that modern RNRs are highly evolved enzymes that have acquired many characteristics since divergence from a common ancestor, we proceed by discussing the path from the urRNR to the modern RNR classes. Furthermore, by defining the urRNR, we can outline the necessary steps on the way from the protoRNR to the urRNR (Figure 4). However, it is important to note that the protoRNR is a hypothesis while the urRNR is a parsimonious reconstruction based on existing enzymes. Since the transitional steps between the hypothetical protoRNR and the reconstructed urRNR are dependent on the hypothesis as well as the reconstruction, we discuss the transition only to clarify the consequences of assuming the hypothesis being true.

**Table 1:** Important traits in modern ribonucleotide reductase (RNR) classes with consequences for a parsimonious reconstruction of the urRNR.

Trait	Class I	Class II	Class III	urRNR
Fold	10-stranded β/α barrel	10-stranded β/α barrel	10-stranded β/α barrel	10-stranded β/α barrel
Substrate	NDP	Either NDP or NTP	NTP	NTP?
Radical generation	Dimetal-oxo center in separate subunit	AdoCbl (B_12_) in enzyme	AdoMet in separate subunit	Metal center in enzyme + dAdo•?
Protein storage radical	Tyrosine in separate subunit	None (AdoCbl regenerated)	Glycine in enzyme	None?
Cysteinyl radical	Yes	Yes	Yes ^(1)^	Likely
Electron and proton-donating cysteine ^(2)^	Yes	Yes	Yes	Yes
Primary reductant	Cysteine pair	Cysteine pair	Cysteine plus formate ^(3)^	Cysteine pair?
Terminal reductant	Thioredoxin, glutaredoxin acting on C-terminal disulfide	Thioredoxin, glutaredoxin acting on C-terminal disulfide	Formate or thioredoxin acting on a disulfide ^(3)^	?
Base	Glutamate	Glutamate	Formate, glutamate ^(4)^	Carboxylate?
Quaternary structure	Homodimer formed between helices A and B ^(5)^	Homodimer formed between helices A and B ^(5,6)^	Homodimer formed between helices A and B ^(5)^	Homodimer formed between helices A and B ^(5)^
Allosteric substrate specificity regulation	Nucleotide binding in dimer interface	Nucleotide binding in dimer interface ^(6)^	Nucleotide binding in dimer interface	Nucleotide binding in dimer interface?
Allosteric activity regulation	47% with ATP-cone ^(7)^	7% with ATP-cone ^(7)^	76% with ATP-cone ^(7)^	Likely not

^(1)^ Two recent independently solved structures of *Thermotoga maritima* class III RNR lack a suitably positioned cysteine [45,46]. Further work is required to determine the mechanistic details of the protein. ^(2)^ The electron and proton-donating cysteine is one of the partners in the cysteine pair working as primary reductant that is only present in class I and II, but see note ^(3)^. ^(3)^ A Cys-Met pair in some class III RNRs is suggested by Wei *et al.* [46,47]. ^(4)^ *Neisseria bacilliformis* E438 suggested by Wei *et al.* [46]. ^(5)^ The dimer geometry is different between class I and II on the one hand and class III RNR on the other, see Section 3.5 and Figure 7. ^(6)^ A monomeric form with an inserted domain mimicking the dimer interface exists [39] (Section 4.3.1). ^(7)^ Activity regulation has only been found in conjunction with an N-terminal ATP-cone. A few RNRs lacking activity regulation due to non-functional ATP-cones are not discriminated by the HMMER profile (Pfam PF03477, see Table 2).

When the crystal structures of representatives from the three classes of RNR were solved [38,39,40], it was clear that all RNRs are homologous. The general fold, the 10-stranded β/α barrel, together with structurally conserved residues, provided convincing evidence for homology. The urRNR was thus a member of the 10-stranded β/α barrel superfamily, to which pyruvate formate lyase-like (PFL-like) proteins also belong. If the general three-dimensional structure is clear, many other traits of the urRNR (Figure 4, Table 1), and in particular its radical generation mechanism, have been debated at length without reaching consensus [41,42,43,44].

### 3.1. Substrate Phosphorylation Level

Modern RNRs differ with respect to the level at which substrates are phosphorylated; substrates are NDPs in all class I and some class II enzymes, and NTPs in the other class II and all class III RNRs. After reduction, dNDPs are phosphorylated to dNTPs by nucleoside diphosphate kinases. The most parsimonious explanation for this pattern is that the urRNR used NTPs and that NDP reduction evolved in class II in the lineage that led to class I RNRs, since, as we will argue for below, class I is derived from class II (Section 4.3.1). Possible selective advantages to NDP reduction are (i) better control of substrate concentrations, since NTPs are abundant in other enzymatic pathways; and (ii) fine-tuning of a common feedback regulation into the more advanced allosteric regulatory mechanisms in operation today (Section 3.5 and Section 3.6).

### 3.2. A Highly Conserved Reaction Mechanism

**Figure 5:**
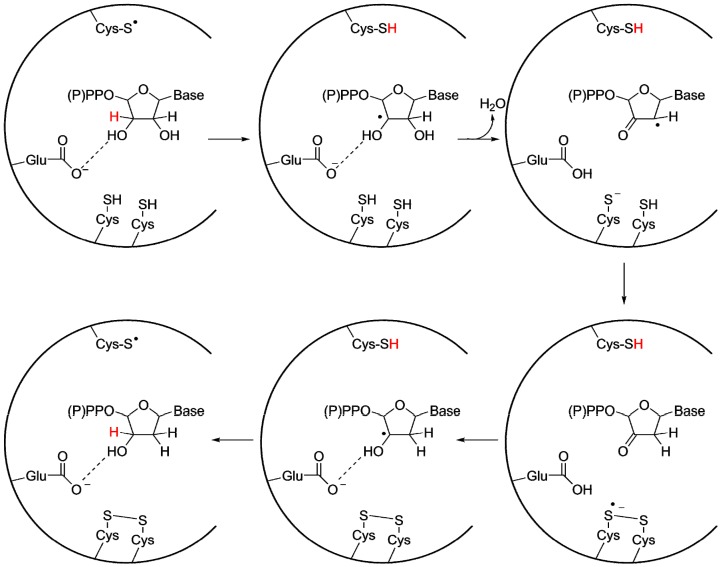
General reaction mechanism for class I and II RNRs, with important side chains in the active site indicated. The radical cysteine (top of the active site) is also present in class III RNR, while the electron donating cysteine pair (bottom of the active site) is replaced in the majority of class III RNRs with a single cysteine. Additionally, in most class III RNRs no residue corresponding to the proton shuttling glutamate has been found, and instead formate has been proposed to fill this function. Figure adapted from reference [55].

Even before structures provided conclusive evidence of the homology of all RNRs, it was still widely believed that the classes were evolutionarily related, since the reaction mechanism is highly similar between all studied RNRs (Figure 5) [48]. A cysteinyl radical activates the substrate by abstracting the 3' H-atom from the ribose on the substrate, resulting in the formation of a 3' carbon radical. In structures of class I, II and III RNRs this cysteine is positioned on a finger loop protruding through the center of the barrel, within van der Waals distance of the substrate’s 3' carbon. After activation and rearrangements, the 2' OH-group leaves as a water molecule, with the proton donated by a second conserved cysteine. A third cysteine, conserved in class I and II RNRs and present in some class III RNRs [46], forms a disulfide bridge with the second cysteine. This cysteine pair acts as an electron relay, providing the reducing equivalents required for the reaction. Finally there is also a number of H-bonding interactions between the protein and the nucleotide, firmly anchoring the phosphate moiety in the active site. The binding to the phosphate groups is likely to have evolved early, as it provides an efficient handle for binding all four substrates in the active site. Crystallographic studies in class I and II RNRs have revealed well-defined interactions between the substrate and a glutamate carboxylate group that is perfectly positioned to act as a base during deprotonation of the 3' OH-group and is involved in proton shuttling during the reaction [49,50,51,52,53]. The ancestry of the glutamate is questionable. It is conserved in both class I and II RNR, but rarely found in class III. For class III, it has been suggested that the electron donating formate also serves as a base during the reaction [54]. Thus the issue whether the protein backbone or a small molecule provided the required base in the urRNR is an open question.

#### Differences Between the Class I/II and the Class III Reaction Mechanisms

Class III RNR differs from class I and II in several ways that have consequences for what may be shared by RNRs by common descent from the urRNR. First of all, the best studied class III RNRs—from *E. coli* [29], Bacteriophage T4 [38,56] and *Lactococcus lactis* [57]—lack the third cysteine and cannot form a di-cysteine disulfide like class I and II RNRs. Instead, they use formate as an electron donor, and it was believed that the reaction proceeds without formation of an intermediate disulfide. However, it was recently proposed that the *E. coli* class III RNR, and by analogy the Bacteriophage T4 and *L. lactis* enzymes, do form a disulfide bridge, but between a cysteine and a methionine [47], opening the possibility that an active site disulfide is a conserved trait in all RNRs. Another trait that differs between class III RNRs on the one hand and the class I and II RNRs on the other is the terminal electron donor. In class I and II, glutaredoxin or thioredoxin acts on an exposed disulfide located on the outside of the protein and formed by two cysteines in the C-terminal part, which in their turn reduce the active site disulfide [58,59]. There is thus an electron transfer pathway between the active site disulfide and the external one. In class III RNRs no external disulfide has been demonstrated, the requisite cysteine residues do not exist, and formate has been shown to act as electron donor [29]. The formate ion also plays the role of base in the class III reaction and coordinator of the 3’ OH-group of the substrate [54]. Wei *et al.* recently suggested that formate is not the reductant in class III RNRs from *N. bacilliformis* and *T. maritima* [46] based on the observation that these organisms lack formate metabolism. Instead, they showed that *N. bacilliformis* class III RNR can use thioredoxin like many class I and II RNRs, and suggest that Glu438 H-bonds with the 3' OH of the substrate and acts as base. Thioredoxin had earlier been shown to have an activating role in the *E. coli* class III RNR [60]. To summarize, we have three different traits that separate many class III RNRs from class I and II, namely the primary and the external electron donors, and the basic carboxylate group that H-bonds to the substrate 3' OH-group. The evolutionary histories of these differences are difficult to disentangle and they thus appear to have evolved together, making it difficult to draw firm conclusions for the urRNR.

Adding further mystery to the class III reaction mechanism, even the unanimous presence of the radical cysteine has been called into question by two recent independent crystal structures of *T. maritima* class III RNR [45,46]. At the position of the canonical radical cysteine is an isoleucine in both structures. The cysteine that is believed to be the radical instigator based on sequence alignments is positioned outside the active site in the crystal structures. Biochemical evidence based on mutation of this cysteine and its neighbor suggest strongly that these residues are found in the active site during catalysis [45], but the conformational changes required to position the radical cysteine near the substrate would be unprecedented in RNRs, so the implications of these structures have still to be unraveled.

### 3.3. Radical-Generation in the UrRNR

The evolutionarily “old” classes of RNR, class II and III, (class I evolved from class II, Section 4.3.1) are different in how the radical is initially formed, and there are thus three possibilities for the nature of this key characteristic of the urRNR: The radical was initiated either by (i) cleavage of AdoMet, as in modern class III; (ii) cleavage of AdoCbl like in modern class II; or (iii) a third mechanism that has not survived. As discussed above, the initial radical in both class II and III is a dAdo•. In the protoRNR we propose that the nucleosyl radical acted directly on the substrate and that the modern mechanism with a cysteinyl radical formed by a side chain of the enzyme was a later development. This cysteine intermediate allowed isolation of the reactive dAdo• species as well as increased steric selectivity of the H-atom transfer reaction by introducing an increased control of its positioning relative to the substrate. After introduction of the cysteine, this mechanism is in principle identical to how modern class II RNR operates. When a substrate binds, AdoCbl is cleaved to form dAdo• that abstracts an H-atom from the cysteine positioned directly adjacent to the 3' position of the substrate ribose [39]. Following the formation of the deoxyribonucleotide product, the catalytic cycle is closed, as AdoCbl is reformed by concerted H-atom transfer via the radical cysteine [61]. Although vitamin B_12_ coenzyme synthesis is a process requiring many steps, it has been suggested that cobalamin is an ancient cofactor [62,63]. Hence, as has been proposed by Poole *et al.* [41], it appears entirely plausible that the class II mechanism of radical generation was present in the urRNR.

To reach the mechanism in class III RNRs, more evolutionary steps are needed. In class III, the dAdo• is not directly involved in abstracting the hydrogen from the radical cysteine. Instead, radical generation is initiated by reductive cleavage of AdoMet by the RNR activase (a maturase of the radical-SAM enzyme family), which in turn stereoselectively abstracts a hydrogen from a specific glycine in the RNR proper (shown in the homologous PFL system [64]). This stable glycyl radical is positioned almost within van der Waals distance of the radical cysteine in the structure [38]. After the Gly• has been formed, the activase is no longer needed since the radical cycles between the glycine, the radical cysteine and the substrate nucleotides [65]. Hence, in class III we have a separation of concerns—radical generation in one protein and ribonucleotide reduction in another—that begs the question of how the separation evolved. Starting from the protoRNR, we can envision two possible evolutionary paths to a class III-like urRNR: (i) The radical generation mechanism specialized to form a separate domain of the protein that was later cleaved off to become the radical-SAM class III activase when direct association was no longer required; or (ii) a radical generation mechanism in the catalytic subunit was replaced by co-option of an already present radical-SAM enzyme able to initiate the radical. While the first alternative, cleavage of an ancestral protein into two separate components, remains a possibility, we find no evidences of this transition in modern RNRs. The fused class III enzymes found in some unicellular eukaryotes clearly have a late origin [66]. Furthermore, before genetic cleavage of the protein, a radical transfer pathway involving the dAdo• plus two amino acids—the storage glycine and the enzymatic cysteine—must have been established. The alternative, co-option of a radical-SAM enzyme, requires either that a suitably positioned glycine was in place to become the storage radical or that the radical-SAM enzyme acted directly on the radical cysteine. Moreover, since the cysteinyl radical is too reactive for storage the radical-generating subunit must in this case remain associated with the reductase to ensure efficient radical generation. Efficiency is, however, likely to be limited, since radical generation by the radical-SAM enzyme is unlikely to be catalytic [19].

In summary, the modern class II radical-generating mechanism, by virtue of its self-sufficiency and biochemical simplicity, appears as the best model for the urRNR. One must keep in mind, however, that both class II and class III RNRs are modern, highly evolved enzymes that may have gained, lost and refined many characteristics since divergence from the urRNR. The radical generation mechanisms in class II and III may thus both be later evolutionary inventions that replaced the ancestral mechanism, but to our knowledge there are no alternatives to AdoCbl and AdoMet as cofactors for this kind of chemistry among modern proteins operating under anaerobic conditions. Other metal centers and organic radicals thus appear more speculative.

### 3.4. The Origin of the 10-Stranded β/α Barrel

All functionally characterized members of the 10-stranded β/α barrel superfamily are GREs or RNRs; class III RNR is both. They are similar in having a reactive cysteinyl radical, situated on a finger loop inserted in the center of the barrel near the substrate, which performs activation of the substrate via H-atom abstraction [48,67,68]. GREs, including class III RNRs, have a glycyl storage radical, formed by the action of a radical-SAM activase, that abstracts the H-atom from the cysteine. Class I and II RNRs generate the radical by different means. Since the glycyl radical is sensitive to oxygen—the peptide chain is cleaved in presence of only trace amounts of oxygen [69,70]—GREs are only encoded by anaerobic microorganisms. Through the action of substrate activation via radical chemistry, they allow difficult oxidations and rearrangements to occur in the absence of oxygen [68].

**Figure 6:**
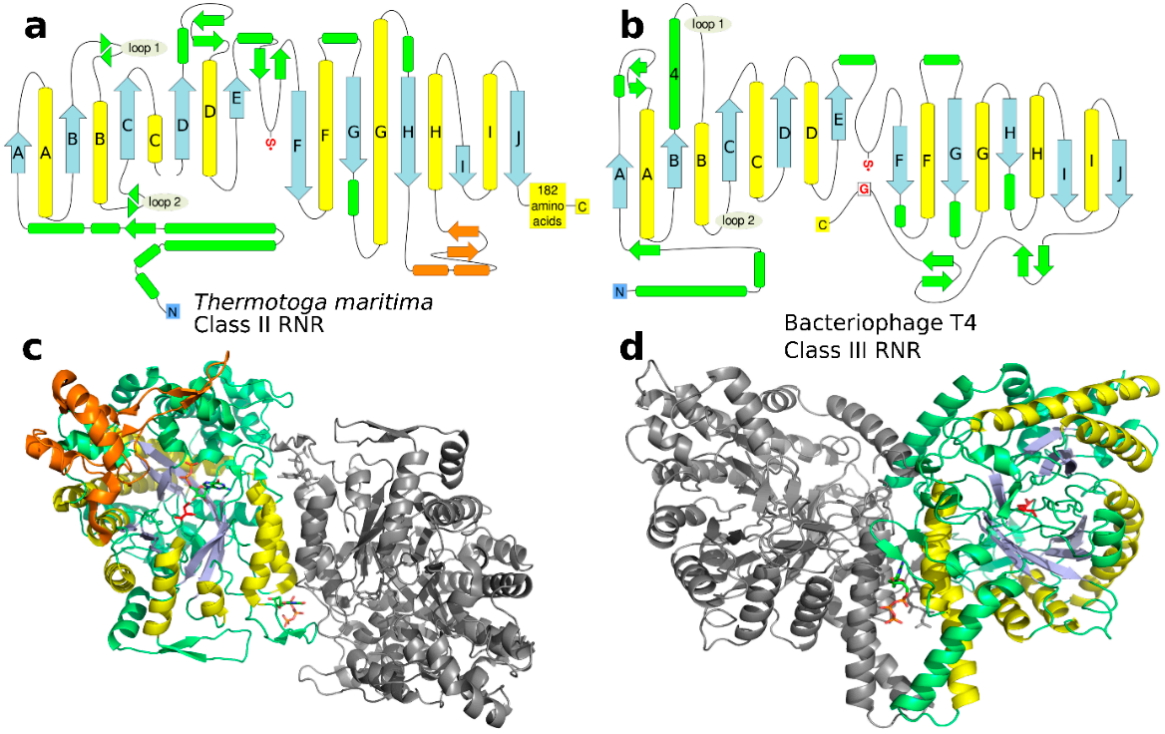
(**a**) and (**b**) Topological diagrams of the class II and class III RNRs from *T. maritima* (PDB: 1XJE [50]) and Bacteriophage T4 (PDB: 1HK8 [38]), respectively. The 10-stranded β/α barrel is blue (β) and yellow (α). Secondary motifs outside the conserved barrel are green, except the B_12_-binding domain in class II, which is orange. The radical cysteine is indicated with a red S• and the radical harboring glycine in class III is indicated by a boxed red G. Loops 1 and 2, implicated in substrate specificity regulation in class II, are indicated in both diagrams; note that only loop 2 is involved in the class III regulation (Section 3.5). (**c**) and (**d**) Structure of the same proteins as in (a) and (b) with substrate specificity effectors bound (dTTP for class II, dGTP for class III; stick representation) and colored like the topological diagrams. The class II structure has a substrate nucleotide (GDP; stick representation) bound in addition to the specificity effector.

Above (Section 2.2.2), we propose a repeated β/α topology for the protoRNR based on that this type of fold is generally considered ancient. Another reason is that the 10-stranded β/α barrel of modern RNRs is also a repeated β/α fold. Topologically, the structure of the 10-stranded β/α barrel consists of two antiparallel halves, each with a (β/α)_5_ configuration (Figure 6). Still it has not, to our knowledge, been suggested to be homologous with the TIM barrel or flavodoxin-like folds. The 8-stranded TIM barrel has been suggested to have evolved from ancestors that only formed half a barrel, *i.e.*, one with four β-strands [71], and a similar argument can be made for the 10-stranded β/α barrel. Presumed gene duplication led to the modern proteins. A possible selective advantage with longer genes coding for the whole protein, is that parts of the barrel are freer to evolve specialized sites for enzymatic activity, substrate binding, *etc.* Conversely, selection would act against longer genes in a small genome with competition for coding space.

The overall structural difference between 8- and 10-stranded β/α barrels is that the 8-stranded TIM barrel is compact, while the 10-stranded RNR/GRE counterpart leaves a cavity in the center of the protein. In RNRs and GREs this cavity forms the substrate-binding pocket, in which the cysteinyl-containing finger-loop is inserted. Starting from a simple cofactor-binding structure consisting of β/α elements and assuming a process whereby β/α elements can be gained, the TIM barrel and the 10-stranded β/α barrel can thus have evolved along similar routes, to reach the modern folds. Different selective pressures as well as other evolutionary contingencies could thus create the TIM barrel where all 8 β-strands are parallel and the 10-stranded β/α barrel where β-strands in the two halves are parallel, but the halves are antiparallel and connected by the cysteinyl radical-bearing finger-loop.

### 3.5. Specificity Regulation—Ancestral or Convergent?

RNR reduces all four ribonucleotides (uracil deoxynucleotides are subsequently converted to thymidine deoxynucleotides by thymidylate synthase; alternatively, thymidine deoxynucleotides are synthesized by deamination of cytosine deoxynucleotides [72]). In modern RNRs, this is accomplished by allosteric regulation of substrate specificity in the enzyme. By competitive binding of effector nucleotides in the specificity site, the specificity of the enzyme is in principle regulated from a substrate whose corresponding deoxyribonucleotide is in excess to one in shortage, to keep all deoxyribonucleotide pools in balance. Modern RNRs are finely tuned in this respect, to counter the mutagenic effect of unbalanced dNTP pools (see [72,73] and references therein).

**Figure 7:**
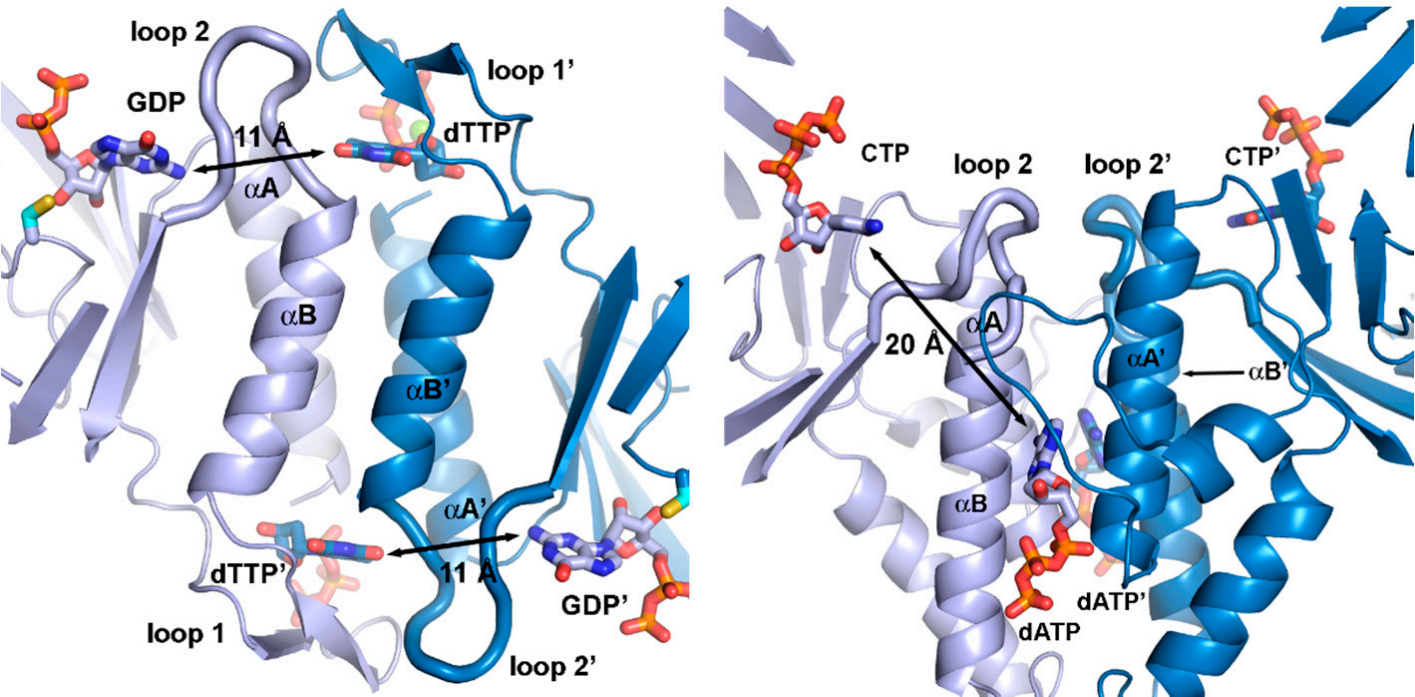
Allosteric substrate specificity effector readout in (**a**) the *T. maritima* class II RNR (PDB: 1XJE [50]) and (**b**) the *T. maritima* class III RNR (PDB: 4COJ [45]). For simplicity, only the specificity site and the strands of each β/α barrel are shown. Effectors (dTTP in (a), dATP in (b)) bind in the dimer interface, although very differently in class II and III due to the different dimerization geometry of the two classes. In class II, effector identity is read out by interactions with loop 1 and 2, while in class III, only loop 2 is involved. Furthermore, the distance from the effector to the active site is almost twice as long (20 Å) in class III as in class II (11 Å).

The effector—dNTP or ATP—binds in the interface between the two subunits of the homodimer formed by a pair of helices in each subunit. The allosteric signal is then transmitted into the active site through subtle conformational changes in a flexible loop known as “loop 2” between two of the β/α-units at the dimer interface (Figure 6 and Figure 7). From comparative studies of the structures of multiple combinations of specificity effectors and their respective substrates bound to class II [50] and class I RNRs [51] a picture has emerged in which the conformational changes in loop 2 result in projection of different combinations of H-bonding groups, either side chain or main-chain, towards the substrate that match the H-bonding pattern and enhance its binding to the active site. In class I and II RNRs there is a clear division of function between the subunits of the dimer regarding effector binding. The effector identity is read out by loop 2 on the same subunit as the affected active site. In parallel, the great majority of interactions with the conserved ribose and triphosphate moieties are handled by the other chain (Figure 6 and Figure 7), mostly by loop 1, which is at the N-terminal end of the same helix that bears loop 2 at its C-terminus. Thus two loops and the two α-helices making up the dimer interface have an intimate interplay, suggesting that binding of the allosteric specificity effector evolved after dimerization.

The specificity site of class III—formed in part by the same two helices, A and B, as in class I and II—is highly divergent from that of the other classes, featuring a relative rotation of one of the subunits by approximately 180° (Figure 6 and Figure 7). The dimer interface also includes interactions between sequence elements N-terminal to the β/α barrel that are unique to class III, making the interface much more extensive in class III. In the specificity site, effector nucleotides bind along the length of the α-helices rather than along their tops. As in class I and II, loop 2 side chains are involved in substrate binding [56], but otherwise the structural basis for specificity regulation is very different. Interestingly, in class I and II RNRs the identity of the effector base is read out solely by H-bonding to main-chain atoms in loop 2, *i.e.*, the readout is independent of the side chains, while in class III it is dependent on conserved side chains in both polypeptides of the dimer. Effector identity is read out by side chains on both of the helices and transmitted indirectly to loop 2 over approximately twice the distance as in the other classes, around 20 Å in class III compared with 11 Å in class I and II (Figure 7).

The origin of the dimer interface geometry in class III is unclear, as the dimerization is different from any other structurally characterized GREs or RNRs. Still, the fact that the dimer interface is made up partly of the same components of the proteins, suggests that dimerization evolved in the urRNR and potentially the allosteric substrate specificity as well. However, the fact that binding of common and variable parts of the effector is more equally distributed between the two chains in class III compared to class I and II, indicates that class III specificity regulation evolved independently from the mechanism in class I and II, although using some of the same structural components. This makes it difficult to draw firm conclusions regarding whether the urRNR had substrate specificity regulation or not.

### 3.6. Activity Regulation—Ancestral and Lost or Multiple Origins?

Overall activity regulation of RNR appears just as important to the organism as the specificity regulation, and the outcome of deviations from the optimum in total dNTP concentration is the same: increased mutational load [72]. In line with this, many RNRs exhibit overall activity regulation by nucleotide-guided allosteric mechanisms in analogy with the specificity regulation [73]. In all cases where overall activity regulation has been studied, competitive binding of ATP and dATP to an N-terminal ATP-cone domain determines enzyme activity. As the ratio of dATP to ATP increases above a certain threshold, the enzyme activity is turned off. It is somewhat enigmatic that activity regulation is not found in all RNRs. Presumably, in organisms encoding RNRs that lack an N-terminal ATP-cone, regulation of the total dNTP concentration takes place transcriptionally.

Recently we conducted an analysis of the presence or absence of ATP-cones in RNRs [74] (summarized at the class level in Table 2). We observe that among closely related RNRs we typically find examples both with and without an ATP-cone. Furthermore, among all three classes of RNRs we find enzymes with more than one N-terminal ATP-cone. We conclude that the distribution is suggestive of a dynamic where ATP-cones are gained and lost in response to selection for control of total dNTP concentrations, rather than the result of ancestral presence and differential loss. But the distribution pattern by itself does not provide conclusive evidence.

**Table 2:** Presence or absence of ATP-cones in the three classes of RNR. RNR sequences where searched with the Pfam [75] ATP-cone HMMER [76] profile: PF03477. Adapted from [74].

RNR class	Frequency of number of ATP-cones (%)				Number of proteins
	0	1	2	3	
I (NrdA/E)	53	33	13	1	4186
II (NrdJ)	93	6	1	0	1800
III (NrdD)	24	70	6	0	2426

Unfortunately, only the most recent RNR class to evolve—class I (Section 4.3.2)—has been studied in any mechanistic detail with respect to activity regulation. Studies in the few class II RNRs with a conserved ATP-cone as well as in class III RNRs with ATP-cones have shown that allosteric regulation in response to different ATP to dATP ratios takes place, but have stopped short of investigating the mechanistic details. This is particularly unfortunate since in class I all studied mechanisms have been found to involve the formation of complexes between the α (catalytic) subunits and the β (radical-generating) subunits [73,74]. The dATP-loaded complexes lack the capacity to form a productive electron transfer chain between the α and β subunits, rendering the enzyme inactive. This interaction has no counterpart, neither in class II nor in class III RNRs, and the mechanism in class I RNR is thus uninformative of the mechanism in the other two classes. By itself, the fact that activity regulation in class I RNR cannot be seen as homologous with activity regulation in the other classes is evidence in favor of the dynamic model of activity regulation evolution and a late origin after the appearance of the urRNR. To get any further into this question, more detailed analyses of the mechanism behind activity regulation within, as well as between, classes of RNRs are necessary.

## 4. Birth of the Three Classes of RNR

As we have mentioned above, modern RNRs are divided into three classes, based on differences in how the cysteinyl radical is formed. We will here attempt to address the question of how the three modern classes evolved after the urRNR from two angles. First we will describe the three classes in further detail to identify characteristics that are likely ancestral or derived. After this we turn to a phylogenetic analysis of RNR classes and evolutionarily related proteins based on solved structures.

### 4.1. Ancestral and Derived Characteristics of the Three RNR Classes

**Figure 8:**
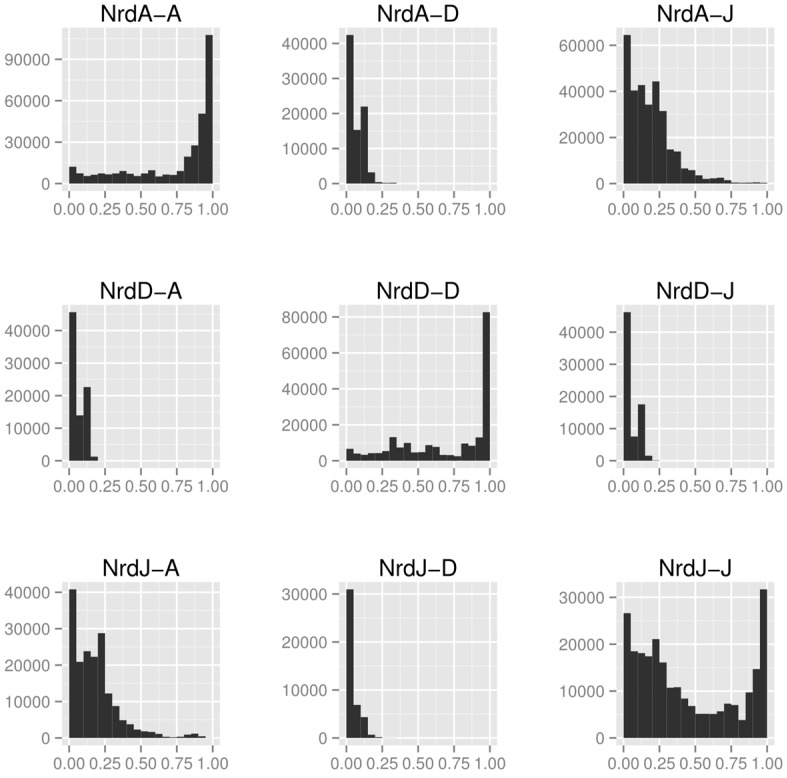
Histograms of alignment to sequence length ratios (x-axes) between the catalytic components of the three RNR classes (NrdA: class I, NrdJ: class II and NrdD: class III). Sequences from the catalytic subunit of all three RNR classes were aligned to each other, within and between classes, using the LAST aligner [77]. In each diagram, the first mentioned enzyme was used as query and the second as target. The different length distributions in the three classes makes the “mirror” diagrams—when a class is used as query and target, respectively—slightly different from each other.

Overall sequence similarity between RNR classes is generally low, here quantified as the ratio of length of best pairwise alignment to length of full sequence (Figure 8). Between classes, class I and II (Figure 8, panels NrdA-J and NrdJ-A) are more similar to each other than either is to class III (Figure 8, panels NrdA-D and NrdJ-D), suggesting the former are more closely related to each other than to class III. Both class I and class III have high within-class similarities but, interestingly, class II does not (Figure 8, panels on the diagonal NrdA-A, NrdD-D and NrdJ-J). The bimodal distribution of sequence similarities in class II is caused by the emergence of the monomeric class II subclass with a dimer-mimicking insertion of 130 amino acids that interrupts alignments [39] (Section 4.3.1).

Although the general sequence similarity between RNR classes is low, certain sequence motifs are conserved in all RNRs or shared between two classes. As discussed above (Section 3.2), several cysteines involved in the reaction mechanism are conserved. The reaction-initiating radical cysteine is present in all known RNRs. Moreover, one (in most class III RNRs) or two (in all class I and II plus some class III RNRs) additional conserved cysteines participate in the reduction of substrates. In all class I and II, a disulfide bridge is formed between the two latter cysteines in the active site (Figure 5). This disulfide is then reduced through the activity of either thioredoxin or glutaredoxin. However, as these reductants cannot be accommodated inside the active site pocket, this disulfide is instead reduced by an electron transfer cascade involving conformational change of another pair of cysteines in the C-terminal part positioned at the surface of the protein, which in turn is reduced by either thioredoxin or glutaredoxin [58,59]. Conversely, in the most thoroughly studied class III RNRs, formate is the reductant [29]. It is believed to act without the involvement of a dicysteine pair. Still, the conserved second cysteine in class III is believed to be involved in electron and proton transfer [47,54] to the 2' C of the substrate (Figure 5). However, as discussed above (Section 3.2), a Met-Cys disulfide bridge and a thioredoxin reductant has been suggested for some class III RNRs [46,47].

**Figure 9:**
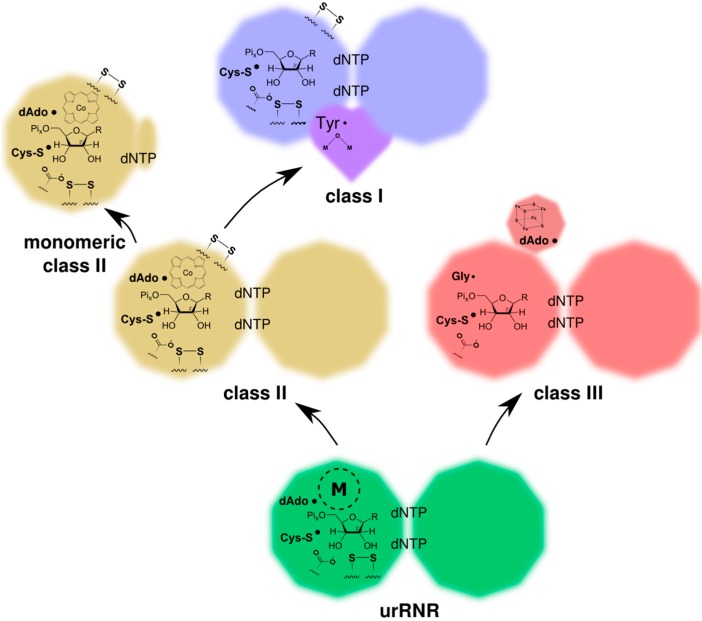
Model for the evolution of the modern three RNR classes from the common ancestor, the urRNR. Informative characteristics for the reconstruction have been indicated. The substrate is shown as a graphical representation of a ribonucleotide while the text “dNTP” in the dimer interface indicates effector nucleotides for substrate specificity regulation (ATP, not being a dNTP, is also an effector). Conserved disulfides in the active site as well as on the outside of the protein in class I and class II, the H-bonding carboxylate/base, and the various radical species are shown. The different radical-generating metal cofactors of the three classes are also shown as well as an “M” within a dashed circle indicating the metal center of the urRNR. See also Table 1.

The presence of two cysteines involved in reduction together with the use of a protein reductant (glutaredoxin or thioredoxin) is thus shared by class I and II but not by a majority of class III RNRs, and when present in class III RNRs the mechanism is different to that in class I and II [46]. Combined, these observations suggest that class I and II are more closely related, as is also indicated by overall sequence similarity between classes. Furthermore, some of the β-strands taking part in B_12_ binding in class II are present in class I RNR [39], but not in class III (Figure 6). In line with this, we present a model for how class II and III diverged from the urRNR, and how class I diverged from class II RNR (Figure 9).

### 4.2. RNR R1/PFL Structural Phylogeny

**Figure 10:**
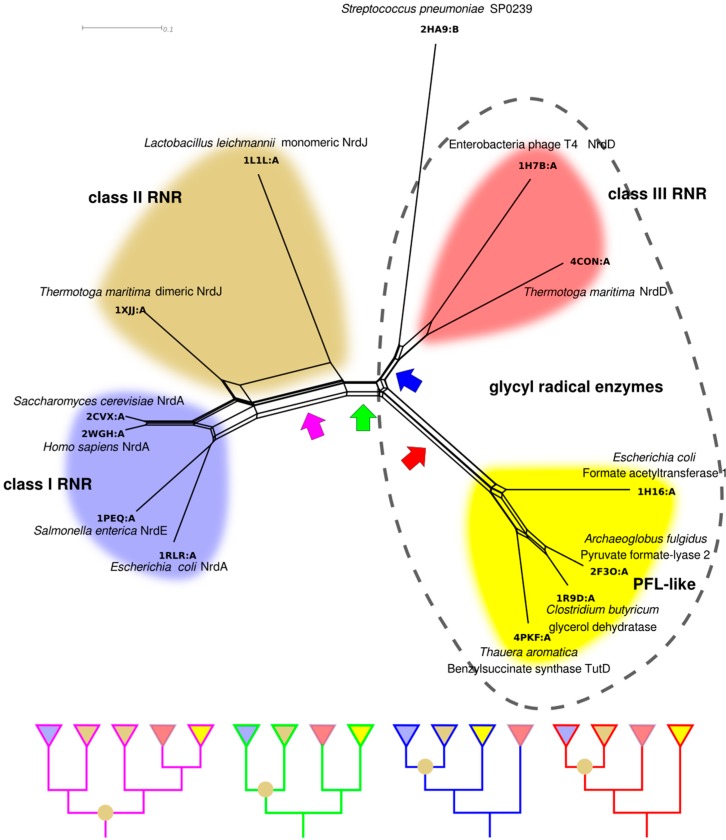
NeighborNet [91] phylogenetic network estimated from pairwise structural distances between representative structures from the 10-stranded β/α barrel superfamily. The phylogeny is presented as a network to illustrate some of the conflicts in the distance matrix. Four potential roots, compatible with monophyly for class I RNR, class III RNR and the PFL-like proteins, are indicated with colored arrows (discussed in the text). Schematic trees, corresponding to each rooting are shown in the panel below. Class II RNR is not monophyletic in any rooting, indicated by an olive circle at an internal node. RNR classes are indicated with colors as in Figure 9, and the PFL-like proteins are in yellow. GREs are indicated by the dashed outline. Structural alignment was performed with GR-Align [92] and the network was constructed with SplitsTree 4 [93], after transforming similarity scores into distances by taking 1 minus the similarity score.

Our model for RNR divergence based on biochemical characteristics (Figure 9) can in principle be tested with phylogenetic methods. However, the low sequence similarities between RNR classes do not allow a simultaneous phylogenetic analysis of all classes with current methodology. Although no explicit evolutionary models for structural evolution exist, structural similarities in the form of phylogenetic networks estimated from structural similarity scores offer an alternative that has been used to glean insight into the evolution of proteins with highly divergent sequences [78,79,80,81,82,83]. Furthermore, structural phylogenetics allows us to include in the analysis other distantly related proteins. Besides several RNR structures in two families “Ribonuc_red_IgC” (class I and II) and “NRDD” (class III), the ECOD [84] “Ten stranded β/α barrel” superfamily (in ECOD termed X-level), contains structures from two non-RNR families: The “PflGly_m” and the “DUF711”. To estimate the phylogenetic network in Figure 10 we selected representatives of all solved structures from the four families. Class I RNRs are well represented with four solved structures of the enzymatic subunit (PDB codes: 1RLR, 1PEQ, 2WGH and 2CVX [40,51,85,86]), while class II (1L1L and 1XJJ [39,50]) and III (1H7B and 4CON [45,56]) have two each. Among the non-RNR enzymes, we identified four structures belonging to the GREs: formate acetyltransferase (1H16 [87]), PFL (2F3O [88]), glycerol dehydratase (1R9D [89]) and benzylsuccinate synthase component TutD (4PKF [90]). The SP0239-like family of unknown function is widespread among *Streptococcus* spp. and is represented by a single solved structure from *Streptococcus pneumoniae* (2HA9, not published) (Figure 10).

In the structure-based phylogenetic network there are splits corresponding to the divergences in our model in Figure 9. There is clear separation of class III from class I and II, consistent with our analysis of class features above. Furthermore, under all reasonable rootings of the network (assuming a root between class I and II or within class I is unlikely), class I is derived from class II; the latter not forming a monophyletic clade without inclusion of class I. Long terminal branches—indicative of a small degree of similarity between a specific protein and all other proteins—are present within both class II and class III RNR, while class I RNR and, to a lesser degree, PFL-like proteins appear more similar within the group. Interestingly, the dimeric class II RNR from *T. maritima* is clearly more similar to class I RNRs than to the monomeric class II RNR from *Lactobacillus leichmannii*, indicated by a strong split separating the two class II structures. Like the bimodal distribution of within-class sequence similarities in class II (Figure 8), this suggests strong divergence within the class, likely following evolution of the dimer-mimicking domain of the *L. leichmannii* class II RNR [39].

Assuming, for the sake of argument, that our structure-based phylogenetic network represents the evolution of the superfamily, it is interesting to consider possible positions for the root and their consequences. Although any split in the network is a possible position for the root, four potential positions for the root appear more likely than the others and have been indicated with colored arrows in Figure 10. Notably, none of them is compatible with monophyly of the RNR classes to the exclusion of all other proteins, and all root positions are compatible with monophyly of class III, class I and the PFL-like proteins. The *purple arrow* indicates a root inside the modern class II diversity. This is consistent with an urRNR not only being similar to class II, but *being* a class II RNR. While an exciting possibility, further structural sampling of class II RNRs is required to investigate the consequences of placing the root here. The *green arrow* splits the network into GREs, including class III RNR, on the one side and non-glycyl radical class II + I RNRs on the other, except for the presence of the SP0239 protein in the GRE clade. The function of SP0239 is not known, but we have not been able to find a potential radical glycine in its amino acid sequence, so it is most likely not a GRE. This root suggests ribonucleotide reduction was the ancestral function of the superfamily, as this is the function present in both halves of the network. The *blue arrow* indicates a root that is consistent with a GRE being ancestral to the whole superfamily and a later appearance of the class II RNR. Furthermore, this root places the PFL-like proteins as a sister group to class I and II RNR, with class III and the SP0239 protein as an outgroup. In other words, this root suggests the unlikely scenario that class II RNR evolved from the PFL-like GREs and not from the class III RNR. The *red arrow* also indicates a root that is consistent with a GRE being the ancestor of the superfamily, but would place the PFL-like proteins as an outgroup to all other proteins. The slight difference between the red and blue roots is likely stretching the power of structural phylogenies too far, especially in light of the functional divergence between RNRs and PFL-like enzymes, and it appears more reasonable to summarize the two into a hypothetical GRE of unknown specificity as potential ancestor of the superfamily.

In summary, shared traits and our structure-based phylogeny support a model of RNR evolution subsequent to the appearance of the urRNR, with class II and III on each side of a divide and class I being derived from class II. Class I is dependent on oxygen and must have evolved after oxygen levels on Earth became sufficiently high, but the relative timing of appearance of class II and III is as yet undecided. We have argued for a homodimeric, self-sufficient RNR similar to class II as the urRNR, but as signs of class III being derived from class II or *vice versa* are lacking, the final word on the nature of radical generation in the urRNR remains to be said.

### 4.3. Selection for the Three Classes and the RNR Repertoire in the Tree of Life

RNRs of all three classes are found in all three domains of the tree of life, plus in many bacterial, archaeal and eukaryotic DNA viruses (Table 3). Many Bacteria and Archaea and some unicellular Eukaryotes encode more than one RNR, often, but not always, from different classes, and mammals encode two radical-harboring class I components (NrdBs, denoted R2 and p53R2). There is, thus, no doubt that there exist selective advantages for the different RNR classes in the modern world, in all likelihood connected to environmental variables such as oxygen presence. We cannot assume, however, that the selective advantage of a certain class in modern organisms is the same as the advantage that drove the diversification of the class from its ancestor.

**Table 3:** Class I RNR proteins are NrdA/E, the reductase, and NrdB/F, the radical-generating subunit; class II is NrdJ and class III is NrdD. Archaea typically encode class II or III, but rarely class I, while Eukaryotes have the opposite pattern. Bacteria encode all three classes, commonly in combination. Several copies of genes encoding the same class are quite commonly found in genomes from all domains. In some organisms, only one of the class I subunits is paralogous. Among the many virus genomes only some dsDNA viruses encode RNR genes. Numbers from RNRdb2 (http://rnrdb.pfitmap.org), counting genomes in the NCBI GenBank with at least one RNR gene.

Domain	Nr genomes	Class I (NrdA/E)	Class I (NrdB/F)	Class II (NrdJ)	Class III (NrdD)
Archaea	117	10	10	72	90
Bacteria	2318	2119	2159	1555	861
Eukaryotes	76	110	129	3	7
Viruses	87	70	51	33	17

#### 4.3.1. Origin of Class II RNR

Above (Chapter 3), we made a parsimonious reconstruction of the last common ancestor of modern RNRs, a single-component urRNR lacking a separate radical-initiating component. The only surviving single-component RNR is the class II, B_12_-dependent RNR. This class is in many ways similar to the urRNR: it does not have a protein storage radical, it uses dAdo• to generate a cysteinyl radical and uses a cysteine pair as primary reducing agent (Table 1). The radical generation in the urRNR is contentious, but since we lean towards a class II-like mechanism (Section 3.3) we propose only refinement of the mechanism along the lineage leading to class II RNR. However, some other traits of class II RNR are likely derived. First among these is the C-terminal cysteine pair, forming a second disulfide bridge that exposes the enzyme to thioredoxin for reduction via an electron transfer cascade [58,59]. The absence of this motif in modern class III RNRs suggests to us that the trait evolved after class II and III separated from their common ancestor, the urRNR. The other alternative, presence of the C-terminal pair of cysteines in the urRNR and loss in class III, appears unlikely given the ubiquitousness of glutaredoxin and thioredoxin systems in organisms. We thus maintain that the C-terminal disulfide was an adaptation in the lineage leading to modern class II RNR. Substrate phosphorylation level is conceivably a clearer example of a derived trait in class II RNR, since the class contains both NDP and NTP reducers. In class III, NTP reduction is omnipresent while class I RNRs exclusively reduces NDPs. Hence, in all likelihood, NDP reduction evolved in class II RNR (see Section 3.1 for a discussion of potential selective advantages of NDP reduction). The last, and probably the least clear, example of a derived class II trait concerns the carboxylate side chain, hydrogen-bonding to the substrate. In our urRNR model we refrain from making a firm suggestion on whether it had a protein-derived carboxylate group, like modern class I and II, or whether a small molecule like the formate found in class III, provided the carboxylate group for substrate hydrogen-bonding. The protein side chain solution appears more advanced and we thus lean towards it evolving along the lineage leading to class II RNR, although we acknowledge that evolution does not always proceed from what we perceive as primitive to advanced.

**Figure 11:**
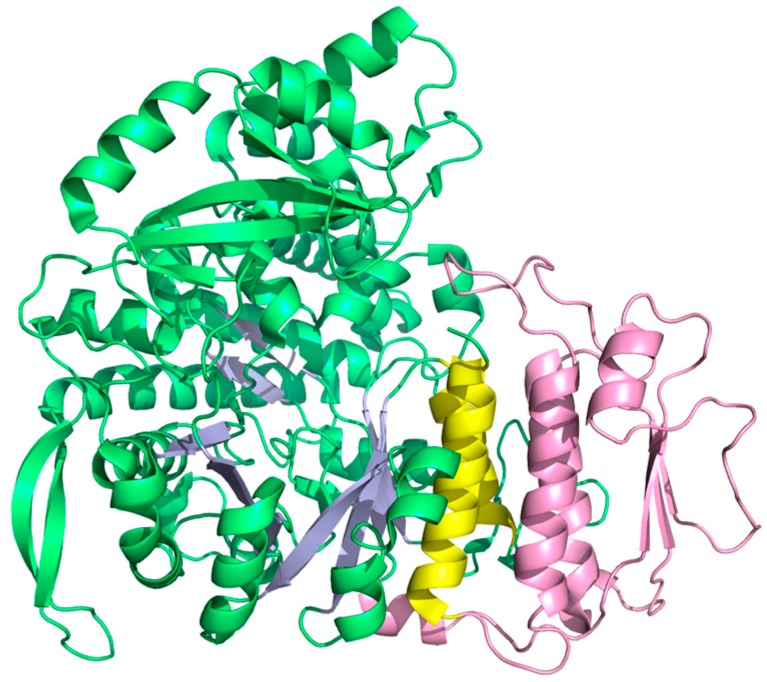
Structure of the monomeric class II RNR from *L. leichmannii* (PDB: 1L1L [39]). Helices A and B are yellow and the dimer-mimicking insertion is pink.

Although class II RNR in many respects appears ancient in comparison with the arguably more sophisticated two component systems with stable protein radicals—class I and III—class II showcases one of the most spectacular evolutionary inventions in RNR posterior to the urRNR and allosteric regulation: The insertion of a dimer-mimicking domain [39], defining a monophyletic subclass encoded by bacterial genomes and a few unicellular Eukaryotes due to horizontal gene transfer [66]. The normal dimer-dependent allosteric substrate specificity mechanism of RNRs closely aligns with models for oligomer-dependent allostery proposed in the mid-1960s [94,95]. Conversely, the monomeric class II RNR is independent of quaternary structure as the effector binds in a pocket formed by helices in a 130 amino acid insertion in the sequence (Figure 11). There are other examples of monomeric enzymes exhibiting allosteric regulation [96,97], but what makes monomeric class II RNR unique is that it evolved from a dimer. It presumably conserves the mechanism from the dimeric enzyme, as suggested by the structural similarity between the dimer interface of class I/II RNR and the pocket formed by the insertion.

#### 4.3.2. Origin of Class I RNR

Class I RNR is an example of selection for the origin of a class not being the same as the likely selection for its fixation in modern organisms. The differences in oxygen requirements exhibited by the class I (dependent on oxygen), class II (independent of oxygen) and class III (poisoned by oxygen) are most likely the reason for the overwhelming number of aerobic bacteria and eukaryotes encoding class I RNR today. In modern aerobic organisms, a class I or II RNR is a requirement and, conversely, under anoxic conditions a class II or III is required. Since class I evolved from the oxygen-independent class II RNR, oxygen-tolerance was not, however, the direct selective advantage of class I at the time of origin. Assuming that class I RNR originated by duplication of a class II RNR gene, the organism where the duplication took place would, by virtue of its class II RNR, already be adapted to oxic environments, although with a B_12_-dependency. Instead, oxygen may have been the enabling factor for a more efficient RNR, as earlier suggested by Poole *et al.* [41] or class I evolved as an escape from cobalt and B_12_-dependency.

The radical-generating R2 subunit of class I RNR (NrdB/F) is a member of the ferritin-like superfamily of proteins [81]. It binds a dinuclear metal center, often diiron, but subclass Ib (NrdF) contains a dimanganese center [98] and subclass Ic contains a heterodinuclear iron manganese center [99,100]. Interestingly, this superfamily is intimately connected with oxygen. The superfamily consists of proteins sharing a fold with a four-helix bundle coordinating a dimetal center. Small members of the superfamily—e.g., ferritin, Dps and rubrerythrin—are just four-helix bundles, although sometimes forming large quaternary complexes (12-meric Dps and 24-meric ferritin balls). The larger members have further structural elements beyond the four-helix bundle.

A plausible scenario for the evolution of the superfamily is that its earliest role in an aerobic environment was to control Fenton chemistry, *i.e.*, the spontaneous reaction of ferrous iron (Fe^2+^) with hydrogen peroxide that produces hydroxyl radicals and oxidized ferric iron (Fe^3+^). Hydroxyl radicals are potent cleavers of DNA and an important reason for why increasing oxygen concentrations were toxic to early life. At a later stage, when concentrations of free ferrous iron, and thus bioavailable iron, had become critically low, due to the oxidation to ferric iron, the fold evolved to become a storage molecule for iron in the form of ferric oxide (Fe_2_O_3_). This is the role played by modern ferritins, bacterioferritins and Dps proteins. But evolution did not stop with control of reactive oxygen species and iron storage, instead a group of enzymes capable of very demanding chemistries evolved [81,101]. In the enzyme family of the ferritin-like proteins, we find the soluble methane monooxygenases [102] catalyzing one of the most demanding chemical reactions in biology—oxidation of methane to methanol—and the radical-generating NrdB/F subunit of class I RNR. In summary, increasing oxygen levels first gave rise to enzymes that reduced the negative impacts of oxygen and later evolved into potent tools for redox chemistry, among them ribonucleotide reduction.

The evolutionary ancestry of the radical-generating subunit of class I RNR suggests that oxygen was important for RNR evolution, not as a way to escape its toxicity, but as a way of harboring its chemical potential. It has, however, not been possible to establish which of the two oxygen-tolerant RNRs (class I and II) is more effective, and it is thus somewhat speculative to suggest that class I evolved for its effectiveness. Besides the possibility of a more effective radical generation mechanism, another potential advantage of class I to class II is its lack of reliance on vitamin B_12_, the biosynthesis of which is complex enough for it not to be encoded by eukaryotes and many bacteria [103]. On the other hand, many eukaryotes, including humans, and possibly bacteria, are dependent on vitamin B_12_ for other enzymatic functions. In summary, while vitamin B_12_-dependency may have played a role in the diversification of class II RNR into class I, it is difficult to forgo the connection with oxygen and the chemical energy available in the combination of iron or manganese with oxygen.

Class I RNR differs from class II also by having a stable protein radical, situated on a tyrosine residue near the metal center in the radical-generating subunit. Although one can easily envisage the advantage of having a non-diffusible radical, it is interesting to note that in subclass Ic the tyrosine is substituted by a phenylalanine and no stable side chain-derived radical is formed but instead a Fe^III^Mn^IV^ center [104,105,106]. At first sight this might appear like a transitional form between an ancestral ferritin-like protein and NrdB, wherein the metal center would be utilized directly as a radical-generator as the stable tyrosyl radical had not yet evolved. This is premature however, since NrdB sequences lacking the radical tyrosine form a tight cluster in phylogenies [66]. Instead, the tyrosyl-free subclass Ic may have evolved as a response to the nitric oxide-sensitivity of the tyrosyl radical (see [104] and references therein); NO is released by the immune system as a response to infection [107]. Even though subclass Ic does not provide us with a transitional form, our conclusion must nevertheless be that radical-generation by the metal center originated prior to the origin of the stable tyrosyl radical. The latter likely evolved under selection for a non-diffusible, stable radical just like the glycyl radical of class III that we turn to in Section 4.3.3.

#### 4.3.3. Origin of Class III RNR and the Glycyl Radical Enzymes

As discussed above (Section 3.3), we find it unlikely that the urRNR exhibited the division of labor exemplified by the modern class I and class III RNRs. Instead, it is easier to envisage that the process leading to class III from a single component urRNR was powered by increased effectiveness of the enzyme after the introduction of a stable amino acid radical in the class III enzymatic subunit (NrdD) and the delegation of the actual radical generation to a separate component (the RNR activase). After activation, *i.e.*, creation of a glycyl radical near the radical cysteine, NrdD is capable of several independent turnovers with the radical cycling between the glycine and the substrate with the radical cysteine as intermediate [65] and is thus safe from transient loss of a cofactor.

If the evidence for what drove the transition from a one-component urRNR to the modern class III RNR is somewhat circumstantial, the origin of the other chemistries exhibited by GREs is even murkier. The small molecule formate—suggested to act as base and reductant in our model protoRNR above—plays a role here. Formate is the reductant in most studied class III RNRs [29] but also the product of the homologous enzyme PFL, another member of the GRE family, which catalyzes the terminal step in fermentation of glucose, formation of acetyl-CoA and formate from pyruvate and coenzyme A [87]. Under anaerobic growth conditions, there is thus a connection between the two chemistries performed by class III RNR and PFL, begging the question: Which was first? This conundrum has been alluded to earlier [41,43,44], but without conclusion. The PFL pathway is not the only anaerobic pathway leading to formate, however. In anaerobic methanotrophs, formate is an intermediate on the way to CO_2_ [108] and conversely, it is a product of CO_2_-reduction by formate dehydrogenases [109]. There are essentially three possible scenarios for the question whether RNR or PFL evolved first: (i) Ribonucleotide reduction was first, performed by an ancestor of class III RNR e.g., a class II-like urRNR, (ii) pyruvate fermentation performed by an ancestor of PFL was first, or (iii) they were simultaneous; the reactions were performed by an unspecific common ancestor. We cannot with certainty rule out any of the alternatives, but each alternative offers some interesting insights into RNR evolution.

If ribonucleotide reduction evolved before pyruvate fermentation to formate, it may be seen as a sign that formate was not used as base or reductant of the urRNR. This is premature, however, since alternative pathways for formate synthesis exists. Syntrophic communities of methanotrophs and anaerobic sulfate-dependent archaeal methane oxidizers may have originated early, providing evidence for an early presence of formate in organisms. However, it seems unlikely that ribonucleotide reduction was confined to these consortia. In the absence of PFL-activity, CO_2_ reduction via formate dehydrogenase thus appears essential to serve as a source of formate in early RNRs if formate was indeed used as reductant. Formate dehydrogenases, FeS-cluster containing or NAD^+^-dependent, have been found in most sequenced organisms (822 archaeal sequences in Uniprot, 78397 bacterial and 936 eukaryotic). Typically, they catalyze the oxidation of formate to CO_2_, but the reaction is reversible and formate should thus in principle be reachable by most organisms through reduction of CO_2_. It is thus quite possible that ribonucleotide reduction preceded pyruvate fermentation while still using formate as base and reductant, and that the fermentative pathway to formate via PFL evolved later.

Turning to the second alternative, that PFL was first, we note that Stubbe *et al.* [43] and Torrents *et al*. [44] used the structural homology of PFL and RNR as an argument for class III RNR being the most ancient of the modern classes. They based their argumentation in part on the assumption that fermentation is an ancient pathway clearly preceding ribonucleotide reduction. This is certainly a possibility, but as far as we can see not decisive evidence. Other fermentative pathways exist, some of them even producing formate, e.g., cleavage of 2-ketobutyrate into propionate and formate in *E. coli* [110]. Furthermore, fermentation requires access to reduced carbon compounds that are used in an energetically ineffective way in the absence of oxidants, and is thus potentially of later origin than DNA and ribonucleotide reduction.

The third alternative, a simultaneous origin of pyruvate fermentation and ribonucleotide reduction, is in line with our model for the protoRNR: An enzyme that acts as a relatively nonspecific substrate activator via radical chemistry allowing energetically demanding reactions to proceed. In PFLs, homolytic cleavage of pyruvate is instigated by activation of the substrate by the cysteinyl radical attacking the carbonyl carbon [68,87]. Notably, our structural phylogenetic network (Figure 10) appears not to support the alternative with a simultaneous origin of pyruvate fermentation and ribonucleotide reduction. However, strong divergent evolution is likely to have reinforced structural differences between enzymes of different function and conversely have acted to homogenize enzymes of similar function, rendering structural comparisons less informative for this question.

## 5. Conclusions

We have painted a picture of the origin and evolution of RNRs, firmly based on evolutionary and biochemical principles, guided by what is observed in modern RNRs and related enzymes. A hypothetical protein with a metal center, the protoRNR, is suggested as the first enzyme capable of ribonucleotide reduction via a general, unspecific H-atom abstraction mechanism. The enzyme was likely not very effective, but given the ubiquitousness of the substrates—the ribonucleotides—in an RNP world, the yield may not have been negligible. From the protoRNR the common ancestor of modern RNRs, the urRNR, evolved driven by selection for a reliable source of deoxyribonucleotides. The urRNR was likely an enzyme without dependence on external enzymes for radical generation, *i.e.*, similar to modern B_12_-dependent class II RNRs. The urRNR was a dimer, which today forms the basis for allosteric substrate specificity regulation, but it was most likely not allosterically activity regulated by means of an ATP-cone. From the urRNR, the three modern classes diverged. Although class II is most similar to the urRNR by being confined to a single component, it still exhibits some derived traits, e.g., the C-terminal cysteine pair involved in reduction of the enzyme and the dimer-mimicking insertion in the monomeric subclass. Class I evolved from class II RNR probably driven by evolutionary discovery of the potent combination of iron and oxygen. Similarly, class III evolved from the urRNR in a process likely driven by selection for more effective ways of generating the essential radical. The stable glycyl radical of class III mirrors the evolution of the radical-generating subunit of class I by supplying a non-diffusible storage point for the radical.

There are many interesting aspects of later RNR evolution that we have not covered in this review, in particular how an essential enzyme like RNR is horizontally transferred between organisms [66] and how this may correlate with environmental factors. We hope to be able to return to that part of the story in the near future.

## Abbreviations

AdoCbl: adenosylcobalamin
AdoMet: *S*-adenosylmethionine
dAdo•: 5'-deoxyadenosyl radical
dNDP: deoxyribonucleoside diphosphate
dNTP: deoxyribonucleoside triphosphate
GRE: glycyl radical enzyme
NDP: ribonucleoside diphosphate
NTP: ribonucleoside triphosphate
NrdA: class I RNR catalytic subunit
NrdB: class I RNR radical generating subunit
NrdD: class III RNR catalytic subunit
NrdE: subclass Ib RNR catalytic subunit
NrdF: subclass Ib RNR radical generating subunit
NrdG: class III RNR activase
NrdJ: class II RNR
PFL: pyruvate formate lyase
RNP: RNA+protein
RNR: ribonucleotide reductase
